# Mutational Profile in Romanian Patients with Hemophilia A

**DOI:** 10.3390/ijms25158366

**Published:** 2024-07-31

**Authors:** Andra Grigore, Mihaela Dragomir, Onda-Tabita Călugăru, Dumitru Jardan, Cerasela Jardan, Melen Brînză, Paul Bălănescu, Daniel Coriu

**Affiliations:** 1 Hematology (Clinic and Laboratory) Discipline-Fundeni Clinical Institute, “Carol Davila” University of Medicine and Pharmacy, 020021 Bucharest, Romania; 2 Department of Hematology and Bone Marrow Transplant, Fundeni Clinical Institute, 022328 Bucharest, Romania; 3 Molecular Biology Laboratory, Medlife, 010093 Bucharest, Romania; 4 Pediatrics Discipline-Fundeni Clinical Institute, “Carol Davila” University of Medicine and Pharmacy, 020021 Bucharest, Romania; 5 Internal Medicine Discipline-Colentina Clinical Hospital, “Carol Davila” University of Medicine and Pharmacy, 020021 Bucharest, Romania

**Keywords:** hemophilia A, F8 gene, next-generation sequencing, novel variant

## Abstract

Hemophilia A (HA) is an X-linked recessive bleeding disorder caused by mutations in the F8 gene, resulting in deficient or dysfunctional factor VIII (FVIII). This study aimed to characterize the mutational profile of HA in Romanian patients using next-generation sequencing (NGS) and multiplex ligation-dependent probe amplification (MLPA). A total of 107 patients were analyzed, revealing pathogenic or likely pathogenic variants in 96.3% of cases. The identified mutations included missense (30.5%), nonsense (9.1%), small deletions (6.4%), small insertions (2.1%), splice-site variants (4.3%), large deletions (1.6%), and large duplications (1.1%). Large intron inversion was previously found in 37.5% of the patients. Novel variants accounted for 21.5% of identified mutations, expanding the spectrum of F8 variants in this population. This study underscores the genetic heterogeneity of HA and provides insights into genotype–phenotype correlations, aiding in clinical management and prenatal diagnosis.

## 1. Introduction

Hemophilia A (HA), an X-linked recessive bleeding disorder, is characterized by a deficiency or dysfunction of clotting factor VIII (FVIII). This genetic disorder affects almost exclusively males, with an incidence of approximately 1 in 5000 live male births worldwide. The clinical phenotype of HA varies from mild to moderate and severe forms depending on the residual coagulant activity levels of FVIII (FVIII:C). Severe HA is characterized by FVIII:C levels below 1%, resulting in spontaneous bleeding episodes. Moderate HA is defined by FVIII:C levels ranging from 1% to 5%, leading to a bleeding tendency even with minor trauma. Patients with mild HA have FVIII:C levels between 5% and 40%, excessive bleeding occurring only in case of hemostatic challenges like trauma or surgery. Hemorrhages predominantly involve the joints and muscles but can also affect internal organs [1].

Factor VIII (FVIII) performs an essential regulatory function within the hemostatic system by enhancing the proteolytic activation of factor X (FX) through activated factor IX (FIXa) on platelet surfaces, during the amplification phase of coagulation [2]. Synthesized primarily in liver sinusoidal endothelial cells [3], FVIII is released as a 2351-amino-acid single-polypeptide chain, from which a 19-amino-acid signal peptide is removed. The mature FVIII protein has the domain structure A1-a1-A2-a2-B-a3-A3-C1-C2 [4]. The heavy chain (A1-a1-A2-a2-B) and the light chain (a3-A3-C1-C2) are non-covalently bound. The heavy chain displays structural heterogeneity due to limited proteolysis occurring within the B domain [5]. B domain plays a significant role in the synthesis, secretion, and stability of FVIII but is not required for its coagulant activity and is cleaved during activation. In the bloodstream, FVIII exists as a heterodimer bound to von Willebrand factor (VWF), which protects it from degradation and helps localize it at sites of vascular injury [6]. Activation of FVIII involves thrombin-mediated cleavage at three specific arginine residues (R372, R740, and R1689), transforming it into an active heterotrimer (A1-a1, A2-a2, A3-C1-C2). This active form plays a critical role in the coagulation process. Deactivation occurs through further cleavage by FX, by activated factor IX (FIX), or by activated protein C, ensuring controlled clot formation [7].

Factor VIII (FVIII) is encoded by the F8 gene, located at the distal end of the long arm of the X chromosome (Xq28). The F8 gene, first cloned in 1984 by Gitschier and colleagues, is structurally complex, spanning 186 kb of genomic DNA and consisting of 26 exons and 25 introns [8]. Mutations in the F8 gene that lead to FVIII deficiency are heterogenous and include point mutations, deletions, insertions, and inversions, with intron 22 and intron 1 inversions being particularly prevalent, especially in severe HA cases. These inversions account for approximately 50% of severe HA cases and 30% of all HA cases [9,10]. Besides large intron inversions, two other significant hotspots have been identified: CpG dinucleotides and stretches of adenine nucleotides [11,12,13].

To date, 3052 unique mutations of various types, excluding inversions, have been documented in the European Association for Haemophilia and Allied Disorders (EAHAD) Coagulation Factor VIII Variant Database—https://f8-db.eahad.org/ (accessed on 12 June 2024). The prevalence of genetic variants in HA includes 66.2% point mutations, 23.4% deletions, 6% duplications, 1.6% insertions, and 1.3% indels, distributed across all exons [14].

The development of neutralizing antibodies (inhibitors) against FVIII complicates treatment, affecting approximately 20–30% of patients with severe HA on replacement therapy during their lifetime and 5–10% of patients at any given time [14,15]. This phenomenon poses substantial clinical challenges by rendering standard treatments ineffective. The risk of inhibitor formation is multifactorial, with genetic factors, particularly the type and localization of F8 mutations, playing pivotal roles [16,17].

In Romania, the national registry for patients with hemophilia is currently under development, thus precluding access to data from this source. According to the 2023 Annual Global Survey Report by the World Federation of Hemophilia, Romania has 1615 individuals diagnosed with HA, with 71 of these patients presenting active inhibitors. Nevertheless, specific data regarding the severity of the disease in this patient population are not available [18]. Moreover, in Romanian patients, the genetic landscape of HA is largely unexplored beyond the known presence of large intron inversions [19]. The current study aims to investigate the mutational profile in a cohort of Romanian HA patients using next-generation sequencing (NGS) and multiplex ligation-dependent probe amplification (MLPA). We expect to expand the spectrum of F8 variants and provide valuable insights for both clinical management of HA patients and prenatal diagnosis in affected families. Characterizing prevalent mutations in this population will enhance our understanding of genotype–phenotype correlations and contribute to more personalized and effective therapeutic approaches.

## 2. Results

Molecular analysis revealed pathogenic or likely pathogenic variants in 103 out of 107 individuals, yielding a variant detection rate of 96.3%. In total, we identified 75 distinct variants, comprising 38 missense variants, 14 nonsense variants, 9 small deletion variants, 4 small duplication variants, 5 splice-site variants, 3 large deletions, and 2 large duplications. A comprehensive summary of the identified F8 gene alterations is provided in Table 1.

**Figure 1:**
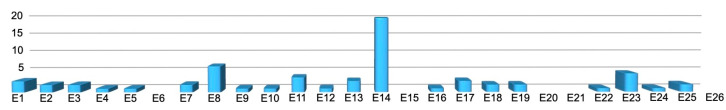
Distribution of variants by exon location.

The distribution of variants spanned all exons of the F8 gene, except for exons 6, 15, 20, 21, and 26 (Figure 1). Mutations were unevenly distributed, with exon 14 harboring the highest proportion (30.8%), followed by exon 8 (10.8%).

Missense variants were the most frequent type of mutation, accounting for 50.67% of the total variant types. Among these, 18 variants were classified as pathogenic and 20 as likely pathogenic. The severity distribution among patients with missense variants showed that 52.7% had severe HA, 33.3% had moderate HA, and 14% had mild HA. Only 3.5% (2/57) of patients with missense variants developed inhibitors, with the mutations occurring in the A3 and C2 domains.

Nonsense variants comprised 18.7% of the identified mutations and were all classified as pathogenic. All patients with nonsense variants exhibited severe HA. Inhibitors were present in 17.6% of these cases (three patients), with the mutations occurring in the B, A3, and C2 domains.

Small deletions constituted 12% of the identified variants, including four single-nucleotide deletions, one two-nucleotide deletion (CT), and two four-nucleotide deletions (TGTT, AAAT). All small deletions were pathogenic, with most patients manifesting severe HA, except for one case with moderate disease. Inhibitors developed in one patient (8.33%) with a mutation in the A3 domain.

Small duplications accounted for 5.33% of the variants. All were pathogenic, and all patients had severe HA. None of the patients with small duplications developed inhibitors.

Splice-site variants made up 6.67% of the identified mutations and were all likely pathogenic. Six patients had severe HA, and two had moderate HA. No inhibitors were detected in these patients.

Three patients with severe HA exhibited large deletions detected by MLPA. These deletions included the first 6 exons, the first 10 exons, and exons 5 and 6, respectively. The deletion of exons 1–10 had not been previously reported. One of these patients developed inhibitors.

Two patients had large duplications detected by MLPA, involving exons 12–14 and 16–22. Both patients had severe HA, and one patient developed inhibitors.

We identified 23 novel variants, including 10 missense variants, 3 nonsense variants, 5 small deletions, 2 splice-site variants, 1 large deletion, and 2 large duplications. All were classified as pathogenic or likely pathogenic, with five novel variants being recurrent. A summary of these variants is provided in Table 1.

**Figure 2:**
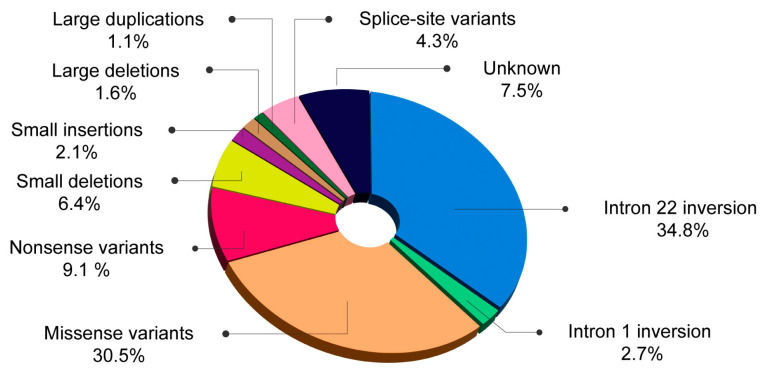
Mutational profile in (%) 187 patients with HA from Romania.

By integrating the results from this study with those of the previously published investigation [19], we examined the mutational profile of 187 patients with HA, comprising 83.4% with severe HA, 12.3% with moderate HA, and 4.3% with mild form of the disease. Large intron inversions were detected in 37.4% of the patients, with inversions in intron 22 occurring in 34.8% and inversions in intron 1 in 2.7%. Missense mutations were identified in 30.5% of the cohort, while nonsense mutations were present in 9.1%. Small deletions were found in 6.4% of patients, small insertions in 2.1%, large deletions in 1.6%, large duplications in 1.1%, and splice-site mutations in 4.3%. The mutational profile remained unknown in 7.5% of the patients: pathogenic variants in the F8 gene were undetectable in four patients (2.1%), while insufficient DNA for further analysis hindered examination in ten patients (5.4%). These findings are depicted in Figure 2.

**Figure 3:**
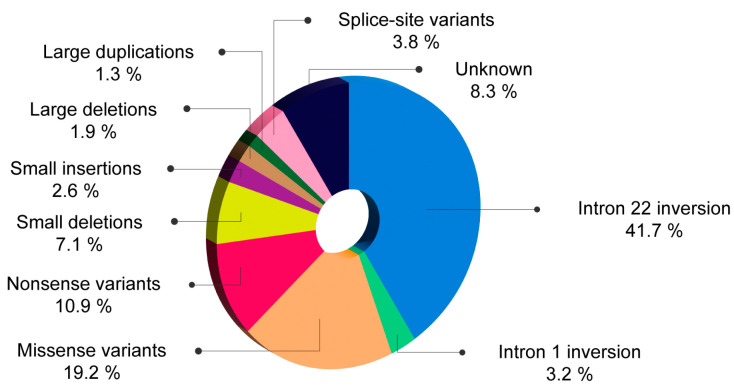
Mutational profile in (%) 156 patients with severe HA from Romania.

In patients with severe HA, the intron 22 inversion emerged as the most prevalent mutation, detected in 41.7% of cases. This was succeeded by missense mutations in 19.2%, nonsense mutations in 10.9%, small deletions in 7.1%, splice-site mutations in 3.8%, intron 1 inversion in 3.2%, small insertions in 2.6%, large deletions in 1.9%, and large duplications in 1.3% of patients. These results are illustrated in Figure 3.

**Figure 4:**
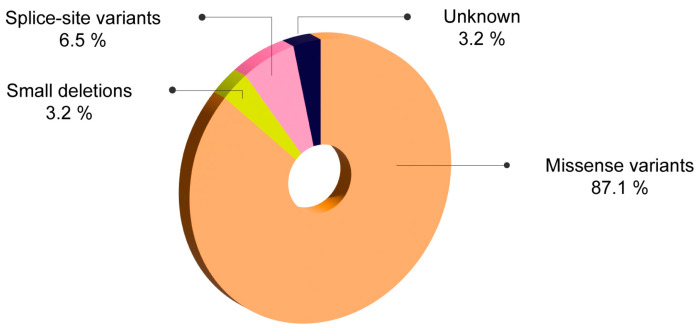
Mutational profile in (%) 31 patients with mild/moderate HA from Romania.

Missense variants were the most prevalent genetic defects in patients with mild/moderate forms of HA, identified in 87.1% of patients. These were followed by splice-site mutations, which were found in 6.5% of cases, and small deletions, detected in 3.2% of cases. These findings are presented in Figure 4.

Overall, inhibitors were detected in 17 patients, constituting 9.09% of the cohort examined. With the exception of one patient who exhibited a moderate form of HA, all patients with inhibitors (94.12%) presented with severe form of the disease. Inhibitors were identified in 10.26% of patients with severe HA and in 3.23% of those with mild to moderate disease. The distribution of inhibitors according to the type of mutation is presented in Table 2. Due to the small sample size, differences in inhibitor occurrence were not statistically significant (*p* = 0.19).

## 3. Discussion

We conducted a comparative analysis of our findings with those from larger studies. Specifically, we compared our results across all severities of HA with the data published by Oldenburg et al. [20], which included 846 individuals with varying HA severities, and by Bach et al. [21], who studied 2671 patients with different HA severities. Additionally, we compared our results in patients with severe HA with those from a meta-analysis by Gouw et al. [22], which encompassed 5383 patients with severe HA, and with a study by Johnsen et al. involving 1272 patients with HA [23]. For mild and moderate HA, we compared our findings with those of Johnsen et al., who analyzed 1048 patients with mild to moderate HA [24]. The comparative data are presented in Table 3. Overall, our results align with these studies. However, some discrepancies, such as the higher prevalence of intron 1 inversion and the increased prevalence of small deletions and insertions in our severe HA cohort, may be attributed to the bias introduced by the smaller number of patients with specific types of mutations.

In total, 21.5% of the identified mutations in the F8 gene are novel, emphasizing the broad spectrum of genetic defects within the F8 gene. Among the newly identified mutations, five are recurrent and may be characteristic of our population. Further investigation is required to validate this hypothesis. The novel c.641T>G variant was found in four unrelated patients with varying HA severities (one patient with severe HA, two patients with moderate disease, and one with a mild form of HA). Two novel variants were discovered simultaneously in one patient, both located in exon 14 and coding for the A2 domain (c.2148T>A and c.2189G>C). Both variants were classified as likely pathogenic. Additional investigation is required to determine the contribution of each variant to the pathogenesis of severe HA. Notably, the identification of 23 novel variants expands the current mutation database, providing new insights into the genetic underpinnings of HA. To the best of our knowledge, this is the first report detailing the mutational profile of Romanian patients with HA.

Most missense variants were located in the A1 and A2 domains (65.8%), with no variant found in the B domain. The A1 and A2 domains play a critical role in FVIII activity and are particularly prone to mutations [24,25]. In contrast, mutations causing HA are rarely found in the B domain, which lacks procoagulant activity and is partially cleaved from the mature FVIII protein [26,27]. Nonsense variants were found in diverse regions, including the signal peptide, a1, A2, B, A3, C1, and C2 domains. The B domain was the most frequent site (28.6%), leading to premature protein termination [10].

Approximately one-third of missense variants (31.58%) and 42.9% of nonsense mutations occurred in CpG dinucleotides coding for arginine. Significantly, the p.(Arg391His) variant was recurrent, being detected in seven unrelated patients. Altogether, CpG dinucleotide hotspots were mutated in 34.6% of the single-nucleotide polymorphisms, our finding being consistent with reports that 40% of point mutations in the F8 gene occur at CpG sites, which comprise only 2% of the coding sequence [28]. Methylation of cytosine at these sites can lead to deamination, converting cytosine to thymidine, which is less efficiently recognized by DNA repair mechanisms. This results in a high mutation rate, particularly impacting arginine residues encoded by CpG-containing codons [11,12].

Small deletions and insertions are frequently observed in repetitive sequences of adenine nucleotides, notably within exon 14 at codons 1191–1194, 1439–1441, and 1588–1590. These mutations arise from polymerase slippage errors during DNA replication or RNA transcription. Such errors may lead to a mitigated phenotype of severe HA with rare bleeding, due to endogenous restoration of the reading frame and subsequent production of low levels of functional FVIII protein [13]. In our study, Poly A-runs were involved in 44.4% of small deletions. In most deletions (five out of nine), the mutation was localized in the region of exon 14, coding for the B domain. All small duplications involved Poly A-runs regions from exon 14, coding for the B domain. Consequently, our findings confirm the existence of a hotspot for F8 mutations within regions containing A-stretches.

No genetic cause was found in 4 cases out of 107: three cases of severe HA and one case with moderate disease. Common inversions, nucleotide substitutions in all F8 gene exons and adjacent intronic regions, large deletions, and insertions were excluded. We cannot rule out deep intronic variants leading to alternative splicing [29], pathogenic variants in regulatory genes, or unique inversions undetectable by NGS and MLPA techniques. HA patients without detectable F8 gene defects may constitute less than 10% of the sample population [30,31,32,33]. Other possible causes include mutations in other genes causing an HA-like phenotype, such as mutations in von Willebrand factor affecting the FVIII binding site in von Willebrand disease (VWD) type 2 Normandy (2N) [34,35], or mutations in the LMAN1 gene (lectin, mannose-binding 1) [36,37] or MCFD2 gene (multiple coagulation factor deficiency 2 protein) [38], leading to combined deficiencies of factor V and FVIII. Further investigation of these patients is necessary to determine the underlying cause of FVIII deficiency.

Mutations that result in the absence of a functional gene product, such as large deletions, large intron inversions, nonsense mutations, non-A-run small deletions, and splice-site mutations, typically manifest as severe HA and increase the likelihood of inhibitor formation. In particular, large deletions spanning multiple domains carry an extremely high risk of developing inhibitors. On the other hand, missense mutations and small deletions/insertions within the A-runs carry a lower risk of inhibitor development, as they may result in the production of an altered but partially functional protein, which can sometimes induce immune tolerance [24,39,40]. In missense mutations, the risk of developing inhibitors depends more on the mutation’s location rather than the severity of the patient’s hemophilia phenotype. Thus, missense mutations located in the light chain pose a significantly higher risk of inhibitor formation, about four times greater compared to mutations elsewhere. In these cases, inhibitor development often occurs later in adulthood and patients typically have a poor response to immune tolerance therapies [24,41].

Garagiola et al. [42] proposed categorizing F8 mutation types into three risk groups: high-risk (large insertions/deletions affecting multiple exons and nonsense mutations on the light chain), intermediate-risk (large insertions/deletions involving a single exon, nonsense mutations on the heavy chain, and large intron inversions), and low-risk (small insertions/deletions, missense mutations, and splice-site mutations). In our cohort, 4.8% (9/187) of patients fell into the high-risk group, 33% of them (3/9) developing inhibitors. The intermediate-risk group comprised 43.3% of patients (81/187), with inhibitors present in 12.3% of them (10/81). The low-risk group included 43.3% of the patients (81/187), with a 3.7% inhibitor rate (3/81).

The patients with large duplications could not be classified in the proposed risk groups. The low prevalence of large duplications might make it difficult to assess the risk of inhibitor development in these patients. In a large study of 2671 patients with HA, large duplications were detected in only 0.64% of the cases [21]. In our study, one of the two patients with large duplications developed inhibitors. Interestingly, the duplicated region (exons 16–22) codes for the light chain. We believe that the hypothesis that duplications in the region encoding the light chain increase the risk of inhibitor development deserves future study in large patient cohorts.

Besides the aforementioned patient with a large duplication in the light chain, five other patients with mutations in the light chain developed inhibitors: two patients with missense variants, two patients with nonsense variants, and one patient with small deletion. Altogether, 35.3% of the patients with inhibitors had mutations in the light chain. Therefore, our study confirms the importance of the light chain in the immunogenicity of FVIII.

The relatively small number of patients with inhibitors in our study limits the statistical power to draw definitive conclusions about the correlation between specific mutations and inhibitor development. Larger cohorts are necessary to validate these findings and to better understand the risk factors associated with inhibitor formation.

The risk stratification of mutations, based on their type and location, provides valuable prognostic information concerning the likelihood of inhibitor formation. This stratification can guide clinical management and therapeutic strategies, optimizing patient outcomes.

Table 4 presents a comparative analysis of the frequency of inhibitor development for each type of mutation in our population, juxtaposed with data from two extensive studies [22,24]. The frequency of inhibitors associated with nonsense mutations, missense variants, and small deletions and insertions aligns with published data. Our findings confirm that inhibitors are more prevalent when mutations are localized in the light chain and when small deletions and insertions occur in regions outside the adenine stretches. Discrepancies in the frequency of inhibitor occurrence were observed in patients with large rearrangements. Specifically, the incidence of inhibitors was lower in those with intron 22 inversion (7 out of 65) and large deletions (one out of three) but higher in those with intron 1 inversion (two out of five). We hypothesize that these variations compared to other studies may be attributable to the limited sample size in our cohort. However, this assumption requires further research involving a larger cohort of patients.

## 4. Materials and Methods

### 4.1. Patients

The study employed a cross-sectional design and encompassed 107 apparently unrelated patients previously diagnosed with HA. These patients successively attended one of the following national centers for hemophilia: Fundeni Clinical Institute, “Prof. Dr. Ion Chiricuta” Institute of Oncology in Cluj Napoca, “Sf. Spiridon” Clinical Emergency Hospital in Iasi, and “Cristian Serban” Medical Center for Evaluation and Rehabilitation for Children in Buzias. We excluded from our study cohort any related patients, as well as 70 patients who tested positive for large intron inversions (intron 22 inversion and intron 1 inversion). Additionally, 10 patients who tested negative for large intron inversions were excluded due to insufficient DNA for further analysis. Among the 107 patients, 71% had severe HA, 21.5% had moderate HA, and 7.5% had mild forms of HA. A family history of HA was reported in 33 cases (30.8%), while 61 patients (57%) had no family history of HA, and in 13 patients (12.2%) the family history was uncertain.

The patient group varied in terms of age and the treatment plans they were following. The mean age was 42 years (SD = 15). The majority of patients were on prophylactic substitution therapy, while 35% received treatment on demand. Patients were systematically screened for inhibitors according to the national protocol for HA, with inhibitors identified in eight patients (7.6%) using the Bethesda assay.

### 4.2. Samples/DNA Extraction

This study was conducted in the Molecular Biology Laboratory of the Hematology Department in Fundeni Clinical Institute. Peripheral whole blood samples were collected in EDTA tubes, followed by genomic DNA extraction using the PureLink Genomic DNA Mini Kit (Invitrogen, Waltham, MA, USA) according to the manufacturer’s instructions. DNA purity and concentration were subsequently assessed using a NanoDrop-1000 spectrophotometer (NanoDrop Technologies, Wilmington, DE, USA).

### 4.3. Next-Generation Sequencing (NGS)

Primers were designed using Primer-BLAST software—https://www.ncbi.nlm.nih.gov/tools/primer-blast/ (accessed on 19 May 2020) to target amplification across all exons and exon–intron junctions of the F8 gene via polymerase chain reaction (PCR). The PCR products were purified using the ProNex^®^ Size-Selective Purification System (Promega, Madison, WI, USA) and subsequently indexed with the IDT for Illumina kit (Illumina, San Diego, CA, USA). The quality and quantity of the samples were assessed using a Qubit™ 4 Fluorometer (Thermo Fisher Scientific, Waltham, MA, USA). Following equimolar pooling of the samples, libraries were sequenced on the Illumina MiSeq platform, entailing a 500-cycle double-indexed paired-end run. For demultiplexing, CASAVA 1.8 software (Illumina, San Diego, CA, USA) was employed. Subsequently, the resulting FASTQ files underwent alignment to the GRCh38 reference genome using DNASTAR Lasergene 17.2 software, followed by visualization using IGV 2.16.0 software. The identified variants were annotated using the Ensembl Database and HGVS nomenclature.

### 4.4. Variant Interpretation

Variant interpretation followed the guidelines of the American College of Medical Genetics (ACMG) [43]. Variants were categorized as pathogenic (P), likely pathogenic (LP), variant of uncertain significance (VUS), benign (B), or likely benign (LB), utilizing 26 criteria of varying strength: 15 pathogenic (PVS1, PS1-4, PM1-6, PP1-4) and 11 benign (BA1, BS1-4, BP1-7 except BP6).

We consulted the ClinVar Database (National Library of Medicine, Bethesda, MD, USA) and the European Association for Hemophilia and Allied Disorders (EAHAD) Factor VIII Gene (F8) Variant Database—https://f8-db.eahad.org/index.php (accessed on 5 May 2024) to ascertain whether the variants had been previously reported. We examined the Genome Aggregation Database (gnomAD)—http://gnomad-sg.org/ (accessed on 5 May 2024) to verify the absence or the low incidence of the variant in the general population. Domain information was retrieved from UniProt—https://www.uniprot.org/ (accessed on 6 April 2024).

### 4.5. Bioinformatic Tools

The potential deleterious effects of the variants were assessed in silico utilizing the following prediction tools: PolyPhen 2.0—http://genetics.bwh.harvard.edu/pph2/ (accessed on 10 June 2024), SIFT—https://sift.bii.a-star.edu.sg/ (accessed on 10 June 2024), and MutationTaster—https://www.mutationtaster.org/ (accessed on 10 June 2024).

### 4.6. MLPA Analysis

For patients with suspected large deletions or duplications, or those who tested negative by NGS, a multiplex ligation-dependent probe amplification (MLPA) assay was performed to detect complex rearrangements in the F8 gene. We utilized the SALSA MLPA Probemix P178-B4 F8 kit (MRC Holland, Amsterdam, The Netherlands) in accordance with the manufacturer’s instructions on a SeqStudio Genetic Analyzer (Applied Biosystems, Waltham, MA, USA). Results were analyzed using Coffalyser.Net software v.240129.1959 (MRC Holland).

### 4.7. Statistical Analysis

The analysis of data was carried out using IBM SPSS Statistics software (version 27) on Mac OS (IBM Corp., Armonk, NY, USA).

## Figures and Tables

**Table 1:** Description of variants in F8 gene identified in this study.

Exon/Intron	Protein Domain	Nucleotide Change	Codon Change	Amino Acid Change	Variant Type	Novel/Known	No of Patients	Phenotype	Inhibitor	Criteria	Classification
E1	A1	c.71A>G	TAC>TGC	p.(Tyr24Cys)	missense	known	1	moderate	no	PM1 PM2 PP2 PP4	LP
E1	A1	c.97T>C	TGG>CGG	p.(Trp33Arg)	missense	novel	1	severe	no	PM1 PM2 PP2 PP4	LP
E2	A1	c.202A>G	ACT>GCT	p.(Thr68Ala)	missense	known	1	severe	no	PM1 PM2 PP2 PP4	LP
E3	A1	c.292G>A	GAG>AAG	p.(Glu98Lys)	missense	known	1	severe	no	PM1 PM2 PP2 PP4	LP
E3	A1	c.326A>G	AAC>AGC	p.(Asn109Ser)	missense	novel	2	severe	no	PM1 PM2 PP1 PP2	LP
E4	A1	c.541G>A	GTG>ATG	p.(Val181Met)	missense	known	3	mild x3	no	PS3 PS4 PM1 PP2	P
E5	A1	c.641T>G	TTT>TGT	p.(Phe214Cys)	missense	novel	4	mild x1/moderate x2/severe x1	no	PM1 PM2 PM5 PP2	LP
E7	A1	c. 871G>A	GAA>AAA	p.(Glu291Lys)	missense	known	1	moderate	no	PS3 PS4 PM1 PP2	P
E7	A1	c.985T>G	TGT>GGT	p.(Cys329Gly)	missense	novel	1	severe	no	PM1 PM2 PM5 PP2	LP
E8	A1	c.1016T>C	ATG>ACG	p.(Met339Thr)	missense	known	1	severe	no	PM1 PM2 PP1 PP2	LP
E8	a1	c.1171C>T	CGC>TGC	p.(Arg391Cys)	missense	known	1	moderate	no	PS3 PS4 PM1 PP2	P
E8	a1	c.1172G>A	CGC>CAC	p.(Arg391His)	missense	known	7	moderatex3/severex4	no	PS3 PS4 PM1 PP2	P
E8	A2	c.1203G>T	TGG>TGT	p.(Trp401Cys)	missense	novel	2	moderate x2	no	PM1 PM2 PP1 PP2	LP
E8	A2	c.1244C>T	GCT>GTT	p.(Ala415Val)	missense	known	1	severe	no	PM1 PM2 PP1 PP2	LP
E9	A2	c.1315G>A	GGT>AGT	p.(Gly439Ser)	missense	known	1	severe	no	PM1 PM2 PM5 PP2	LP
E10	A2	c.1492G>A	GGA>AGA	p.(Gly498Arg)	missense	known	1	moderate	no	PS3 PS4 PM1 PP2	P
E11	A2	c.1595G>C	TGG>TCG	p.(Trp532Ser)	missense	novel	1	severe	no	PM1 PM2 PP2 PP4	LP
E11	A2	c.1648C>T	CGC>TGC	p.(Arg550Cys)	missense	known	1	mild	no	PS3 PS4 PM1 PP2	P
E11	A2	c.1683T>G	GAT>GAG	p.(Asp561Glu)	missense	novel	1	moderate	no	PM1 PM2 PM5 PP2	LP
E13	A2	c.1909A>G	AAT>GAT	p.(Asn637Asp)	missense	known	1	severe	no	PM1 PM2 PM5 PP2	LP
E13	A2	c.1990C>G	CAG>GAG	p.(Gln664Glu)	missense	novel	1	severe	no	PM1 PM2 PP1 PP2	LP
E13	A2	c.2048A>G	TAT>TGT	p.(Tyr683Cys)	missense	known	3	severe x3	no	PS3 PS4 PM1 PP2	P
E14	A2	c.2148T>A	TTT>TTA	p.(Phe716Leu)	missense	novel	1	severe	no	PM1 PM2 PP1 PP2	LP
E14	A2	c.2149C>T	CGG>TGG	p.(Arg717Trp)	missense	known	1	moderate	no	PS3 PS4 PM1 PP2	P
E14	A2	c.2150G>A	CGG>CAG	p.(Arg717Gln)	missense	known	1	moderate	no	PS3 PS4 PM1 PP2	P
E14	A2	c.2167G>A	GCC>ACC	p.(Ala723Thr)	missense	known	1	mild	no	PM1 PM2 PM5 PP2	LP
E14	A2	c.2189G>C	TGT>TCT	p.(Cys730Ser)	missense	novel	1	severe	no	PM1 PM2 PP1 PP2	LP
E14	a3	c.5122C>T	CGC>TGC	p.(Arg1708Cys)	missense	known	2	mild/moderate	no	PS3 PS4 PM1 PP2	P
E16	A3	c.5398C>T	CGT>TGT	p.(Arg1800Cys)	missense	known	1	severe	no	PS3 PS4 PM1 PP2	P
E17	A3	c.5593G>T	GAT>TAT	p.(Asp1865Tyr)	missense	known	1	severe	no	PS3 PS4 PM1 PP2	P
E17	A3	c.5815G>A	GCA>ACA	p.(Ala1939Thr)	missense	known	1	severe	no	PM1 PM2 PM5 PP2	LP
E18	A3	c.5824G>A	GGC>AGC	p.(Gly1942Ser)	missense	known	1	severe	no	PM1 PM2 PP2 PP4	LP
E19	A3	c.6046C>T	CGG>TGG	p.(Arg2016Trp)	missense	known	4	moderate x3/severe x1	yes (1 patient)	PS3 PS4 PM1 PP2	P
E23	C1	c.6506G>A	CGT>CAT	p.(Arg2169His)	missense	known	1	mild	no	PS3 PS4 PM1 PP2	P
E23	C1	c.6533G>A	CGC>CAC	p.(Arg2178His)	missense	known	1	moderate	no	PS1 PS3 PS4 PM1 PP2	P
E23	C1	c.6544C>T	CGC>TGC	p.(Arg2182Cys)	missense	known	1	severe	no	PS3 PS4 PM1 PP2	P
E23	C1	c.6545G>A	CGC>CAC	p.(Arg2182His)	missense	known	2	severe x2	no	PS3 PS4 PM1 PP2	P
E24	C2	c.6683G>A	GGA>CAA	p.(Arg2228Gln)	missense	known	1	severe	yes	PS1 PS3 PS4 PM1 PP2	P
E1	signal peptide	c.4C>T	CAA>TAA	p.(Gln2*)	nonsense	novel	1	severe	no	PVS1 PM1 PM2 PM4 PP1	P
E8	a1	c.1063C>T	CGA>TGA	p.(Arg355*)	nonsense	known	1	severe	no	PVS1 PM1 PM2 PM4	P
E8	A2	c.1203G>A	TGG>TGA	p.(Trp401*)	nonsense	known	1	severe	no	PVS1 PM1 PM2 PM4 PP1	P
E11	A2	c.1750C>T	CAG>TAG	p.(Gln584*)	nonsense	novel	1	severe	no	PVS1 PM1 PM2 PM4	P
E12	A2	c.1804C>T	CGA>TGA	p.(Arg602*)	nonsense	known	1	severe	no	PVS1 PM1 PM2 PM4	P
E14	B	c.2440C>T	CGA>TGA	p.(Arg814*)	nonsense	known	2	severe x2	no	PVS1 PM1 PM2 PM4	P
E14	B	c.3380G>A	TGG>TAG	p.(Trp1127*)	nonsense	novel	2	severe x2	no	PVS1 PM1 PM2 PM4 PP1	P
E14	B	c.4128C>A	TAC>TAA	p.(Tyr1376*)	nonsense	known	1	severe	no	PVS1 PM1 PM2 PM4 PP1	P
E14	B	c.4408G>T	GAG>TAG	p.(Glu1470*)	nonsense	known	1	severe	yes	PVS1 PM1 PM2 PM4	P
E17	A3	c.5766C>A	TGC>TGA	p.(Cys1922*)	nonsense	known	1	severe	no	PVS1 PM1 PM2 PM4	P
E18	A3	c.5878C>T	CGA>TGA	p.(Arg1960*)	nonsense	known	1	severe	yes	PVS1 PM1 PM2 PM4 PP1	P
E22	C1	c.6403C>T	CGA>TGA	p.(Arg2135*)	nonsense	known	2	severe x2	no	PVS1 PM1 PM2 PM4 PP1	P
E23	C1	c.6496C>T	CGA>TGA	p.(Arg2166*)	nonsense	known	1	severe	no	PVS1 PM1 PM2 PM4	P
E25	C2	c.6743G>A	TGG>TAG	p.(Trp2248*)	nonsense	known	1	severe	yes	PVS1 PM1 PM2 PM4 PP1	P
E2	A1	c.206_209delTGTT		p.(Leu69Leufs*2)	frameshift	novel	1	severe	no	PVS1 PM1 PM2 PM4 PP1	P
E14	a2	c.2218delG		p.(Asp740Thrfs*11)	frameshift	novel	1	severe	no	PVS1 PM1 PM2 PM4	P
E14	B	c.3637delA		p.(Ile1213Phefs*5)	frameshift	known	3	severe x3	no	PVS1 PM1 PM2 PM4 PP1	P
E14	B	c.4085delA		p.(Asn1362Thrfs*1)	frameshift	novel	1	severe	no	PVS1 PM1 PM2 PM4	P
E14	B	c.4370delC		p.(Ala1457Alafs*8)	frameshift	novel	1	moderate	no	PVS1 PM1 PM2 PM4	P
E14	B	c.4372delA		p.(Lys1458Lysfs*7)	frameshift	known	2	severe x2	no	PVS1 PM1 PM2 PM4	P
E14	B	c.4377_4380delAAAT		p.(Lys1459Lysfs*4)	frameshift	novel	1	severe	no	PVS1 PM1 PM2 PM4	P
E19	A3	c.6082delG		p.(Met2029*)	frameshift	known	1	severe	yes	PVS1 PM1 PM2 PM4	P
E25	C2	c.6876_6877delCT		p.(Phe2294Serfs*90)	frameshift	known	1	severe	no	PVS1 PM1 PM2 PM4	P
E14	B	c.2945dupA		p.(Asn982Lysfs*9)	frameshift	known	1	severe	no	PVS1 PM1 PM2 PM4 PP1	P
E14	B	c.3300dupA		p.(Glu1101Argfs*16)	frameshift	known	1	severe	no	PVS1 PM1 PM2 PM4 PP1	P
E14	B	c.3637duplA		p.(Ile1213Asnfs*28)	frameshift	known	1	severe	no	PVS1 PM1 PM2 PM4	P
E14	B	c.4379dupA		p.(Asn1460Lysfs*1)	frameshift	known	1	severe	no	PVS1 PM1 PM2 PM4 PP1	P
I6		c.787+3A>G			splice	known	3	severe x3	no	PPM1 PM2 PP1 PP4 PP5	LP
I9		c.1444-1G>A			splice	novel	1	severe	no	PM1 PM2 PP1 PP4	LP
I11		c.1753-2A>C			splice	known	1	severe	no	PM1 PM2 PP1 PP4	LP
I11		c.1753-12T>A			splice	novel	2	moderate x2	no	PM1 PM2 PP1 PP4	LP
I17		c.5816-2A>G			splice	known	1	severe	no	PPM1 PM2 PP1 PP4 PP5	LP
		Del. Ex. 1-6			deletion	known	1	severe	yes		
		Del. Ex. 1-10			deletion	novel	1	severe	no		
		Del ex.5-6			deletion	known	1	severe	no		
		Dupl. ex. 2-14			duplication	novel	1	severe	no		
		Dupl. ex. 16-22			duplication	novel	1	severe	yes		

Abbreviations: E, exon; I, intron; LP, likely pathogenic; P, pathogenic.

**Table 2:** Distribution of inhibitors according to the type of mutation.

Mutation Type	Inversion 22	Inversion 1	Missense	Nonsense	Small Deletions	Small Duplications	Splice-Site Variants	Large Deletions	Large Duplications	Unknown	Total
Number of patients	65	5	57	17	12	4	8	3	2	14	187
Patients with inhibitors	7	2	2	3	1	0	0	1	1	0	17
Inhibitor frequency	10.77%	40%	3.51%	17.65%	8.33%	0%	0%	33.33%	50%	0%	9.09%

**Table 3:** Comparison of mutation frequencies in Romanian HA patients and other populations.

	HA-All Severities			Severe HA			Mild/Moderate HA	
Mutation Type	Intron 22 Inversion	Intron 1 Inversion	Missense Variants	Nonsense Variants	Small Deletions and Insertions	Large Deletions	Large Duplications	Splice-Site Mutations
Romanian patients (n = 187)	34.8%	2.7%	30.5%	9.1%	8.5%	1.6%	1.1%	4.3%
Oldenburg et al. [21] (n = 846)	35.7%	1%	38.2%	9.3%	10.1%	3%	0%	2.6%
Bach et al. [22] (n = 2671)	25.38%	1.12%	43.77%	7.08%	10.71%	2.81%	0.64%	3.59%
Romanian patients (n = 156)	41.7%	3.2%	19.2%	10.9%	9.7%	1.9%	1.3%	3.8%
Gouw et al. [23] (n = 5383)	45%	2%	15%	10%	16%	3%	0%	3%
Johnsen et al. [24] (n = 1272)	42%	1.4%	17.4%	10.7%	17.4%	NA	NA	3.3%
Romanian patients (n = 31)	0%	0%	87.1%	0%	3.2%	0%	0%	6.5%
Johnsen et al. [24] (n = 1048)	3.7%	0.2%	79.5%	1.1%	2%	NA	NA	2.7%

NA—not available.

**Table 4:** Type of F8 gene mutation and inhibitor development in Romanian patient with HA versus other populations.

Mutation Type	Inhibitor Frequency in Romanian Patients (n = 187)	Inhibitor Frequency-Gouw et al. [22] Meta-Analysis (n = 922)	Inhibitor Frequency-Oldenburg et al. [24] (Bonn Centre Study + HAMSTERS Databese)
Intron 22 inversion	10.77%	NA	21%
Intron 1 inversion	40%	NA	17%
Nonsense mutations in the light chain	33.33%	43%	40%
Nonsense mutations outside the light chain	9.09%	12%	17%
Missense mutations in the light chain	14.3%	11%	10%
Missense mutations outside the light chain	0%	6%	3%
Small deletions and insertions in poly-A runs	0%	6%	3%
Small deletions and insertions outside poly-A runs	16.67%	19%	21%
Large deletions > 1 exon	33.33%	67%	88%
Splice-site mutations	0%	7%	17%

NA—not available.

## Data Availability

Data used in this study may be provided by the corresponding author upon reasonable request.
